# Connecting physical cues and tissue patterning

**DOI:** 10.7554/eLife.11375

**Published:** 2015-10-16

**Authors:** Damian Dalle Nogare, Ajay B Chitnis

**Affiliations:** Section on Neural Developmental Dynamics, Eunice Kennedy Shriver National Institute of Child Health and Human Development, Bethesda, United Statesdalledam@mail.nih.gov; Section on Neural Developmental Dynamics, Eunice Kennedy Shriver National Institute of Child Health and Human Development, Bethesda, United Stateschitnisa@mail.nih.gov

**Keywords:** Amotl2, Hippo, proliferation, organ size, Yap1, Lef1, zebrafish

## Abstract

Several signaling pathways work together, via a protein called Amotl2a, to establish the size and shape of a zebrafish sense organ primordium.

**Related research article** Agarwala S, Duquesne S, Liu K, Boehm A, Grimm L, Link S, König S, Eimer S, Ronneberger O, Lecaudey V. 2015. Amotl2a interacts with Hippo effector Yap1 and Wnt/ß-catenin effector Lef1 to control tissue size in zebrafish. *eLife* **4**:e08201. doi: 10.7554/eLife.08201

**Image** When zebrafish lack a protein called Amotl2a (bottom), the posterior Lateral Line Primordium increases in size and cell number

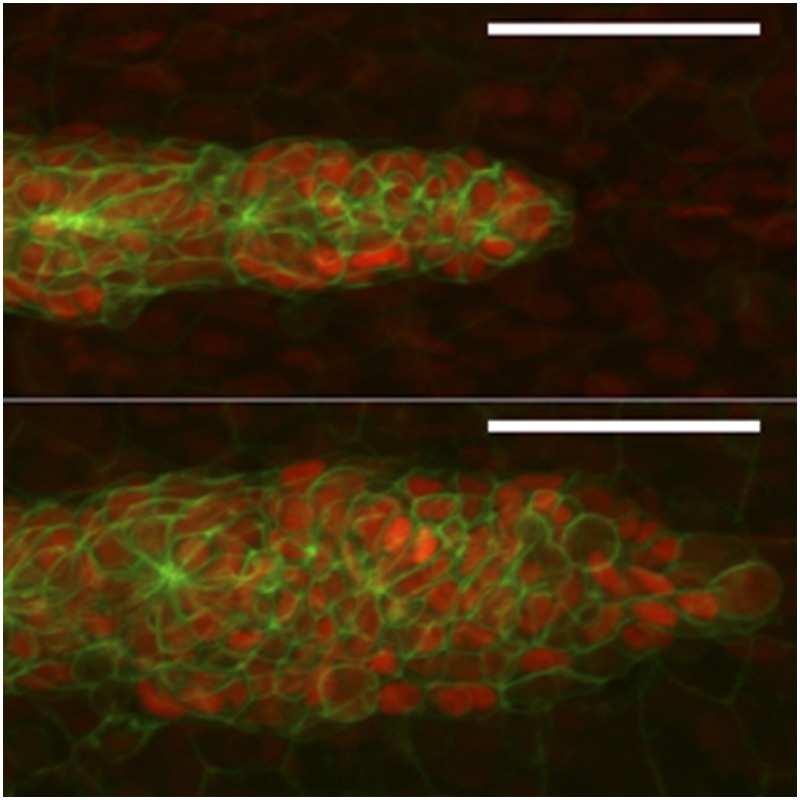

How do local interactions collectively determine the global properties of an organ, like its size and shape, in a developing embryo? Cells can alter how they grow and divide in response to a number of cues, including the mechanical tension between cells and how tightly packed the cells are, via a system called the Hippo signaling pathway (Shroeder and Halder, 2012). Now, in eLife, Virginie Lecaudey and colleagues at the Albert Ludwigs University of Freiburg – including Sobhika Agarwala as first author – have examined how a protein called Amotl2a links the Hippo pathway to other signaling pathways as the zebrafish posterior Lateral Line Primordium develops (Agarwala et al., 2015; Figure 1). This Primordium is the forerunner of a system of sense organs called the posterior Lateral Line that detects the flow of water over the zebrafish (Chitnis et al., 2012).

**Figure 1.**
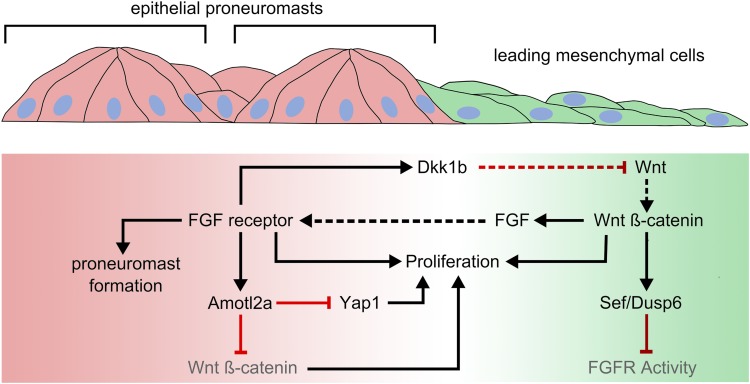
Amotl2a expression limits growth in the posterior Lateral Line Primordium by inhibiting Yap1 and ß-catenin-dependent proliferation. Wnt signaling dominates in a leading zone (green), while FGF receptor activity dominates in a trailing zone (red). Wnt/ß-catenin signaling drives the expression of secreted FGF ligands; it also prevents a response to FGF in the leading cells by triggering the expression of Sef and Dusp6, inhibitors of FGF receptor activation. Activation of the FGF receptor in trailing cells causes proneuromasts to form. It also determines the expression of a diffusible antagonist of Wnt signaling, Dkk1b, which helps restrict Wnt/ß-catenin signaling to a progressively smaller leading zone. Both Wnt and FGF signaling promote cell proliferation in the posterior Lateral Line Primordium. FGF-dependent Amotl2a expression in the trailing zone inhibits proliferation determined by Yap1 and by ß-catenin. Black and red lines represent positive and negative regulatory interactions, respectively. Solid arrows represent intracellular regulatory interactions, while dashed lines represent interactions mediated by secreted factors.

The posterior Lateral Line Primordium starts as a group of about 125 cells that migrate collectively (under the skin) from the ear of the developing zebrafish to the tip of its tail. While the leading cells are relatively mesenchymal, the trailing cells organize into clusters (or “rosettes”) called proneuromasts. These are deposited at regular intervals as the Primordium moves towards the tail. The five or six proneuromasts deposited by the Primordium will develop into the neuromasts that act as the sensory organs of the posterior lateral line. Cells that are not incorporated into proneuromasts are deposited between neuromasts as so-called interneuromast cells. The Primordium shrinks as it moves, and resolves into two or three terminal neuromasts when it finally reaches the tail.

The Wnt and FGF signaling systems coordinate the development of the posterior Lateral Line Primordium. FGF signaling promotes the formation of proneuromasts. In the leading cells, the activity of a Wnt signaling molecule called ß-catenin drives the expression of FGFs (Fibroblast Growth Factors) that activate FGF signaling by binding to their receptor proteins (Figure 1). However, this ß-catenin activity simultaneously inhibits the FGF receptors in the leading cells. As a result, only the trailing cells, where Wnt signaling is weaker, respond to FGF signaling and so form proneuromasts. FGF signaling also drives the expression of a diffusible antagonist molecule that blocks the receptors that are needed for Wnt signaling. This helps to restrict the area of active Wnt signaling to a progressively smaller leading zone. In turn, this allows more of the posterior Lateral Line Primordium to respond to FGF signaling, and so additional proneuromasts form closer to the leading edge.

The transcriptional co-activators YAP and TAZ promote tissue growth and play a central role in another signaling system called the Hippo pathway (Varelas, 2014). Proteins called Motins provide a critical link between external cues, like cell density, and the activity of YAP (Moleirinho et al., 2014). Motins can associate with structural elements of the cell (such as the “tight junctions” that connect neighboring cells) and with YAP. It is thought that cues such as changes in cell–cell adhesion influence how well YAP associates with Motin proteins, which in turn regulates Hippo signaling and hence cell growth. However, the mechanisms behind this remain unclear.

Agarwala et al. show that a Motin called Amotl2a is expressed in the trailing zone of the posterior Lateral Line Primordium in response to FGF signaling. Further investigation revealed that Yap1 and Taz (the zebrafish versions of YAP and TAZ) strongly interact with Amotl2a, and the loss of Yap1 reduces the number of cells in the posterior Lateral Line Primordium. By contrast, the loss of Amotl2a increases cell proliferation. This led Agarwala et al. to hypothesize that the increased proliferation in mutant fish that lack Amotl2a is due to the effects of unregulated Yap1. However, the hyperproliferation observed in Amotl2a mutants is only partially rescued in mutant zebrafish that lack both Amotl2a and Yap1, and remains higher than in mutants that lack only Yap1. This suggests that the increased number of posterior Lateral Line Primordium cells seen in Amotl2a mutants cannot be accounted for by increased Yap1-dependent proliferation.

As Wnt signaling is also known to regulate cell proliferation in the posterior Lateral Line Primordium, Agarwala et al. looked at the pattern of Wnt activation in the Amotl2a mutants and found it was significantly expanded. Furthermore, they found that blocking the Wnt signaling pathway in Amotl2a/Yap1 double mutants reduced cell proliferation to levels seen in Yap1 mutants. This, together with previous studies that have shown that Amotl2a interacts with ß-catenin, led Agarwala et al. to conclude that FGF-dependent Amotl2a expression limits growth in the Primordium by inhibiting Yap1 and ß-catenin-dependent proliferation.

FGF signaling promotes both proliferation and morphogenesis of epithelial rosettes. In this context, Amotl2a expression, which is also dependent on FGF, could be part of a feedback system that limits growth in maturing proneuromasts. However, the loss of Amotl2a does not result in either larger neuromasts or more intervening interneuromast cells. Instead, it results in the deposition of an additional neuromast, consistent with previous studies suggesting that deposition is regulated by posterior Lateral Line Primordium growth (Aman et al., 2011). The migration of the Primordium is also slower in Amotl2a mutants, which could contribute to the deposition of the additional neuromast. This raises the possibility that, as has been suggested in other contexts (Das et al., 2015), Amotl2a also contributes to effective collective migration. By inhibiting Wnt/ß-catenin signaling, Amotl2a may also regulate the pace at which the Wnt system shrinks and the proneuromasts form.

While the Agarwala et al. paper does not, as yet, provide definitive answers, it opens the door to the investigation of these interesting possibilities, and provides a new key to understanding the connection between physical cues and tissue patterning.
